# Health-seeking behaviours of people with osteoarthritis in sub-Saharan Africa: A scoping review

**DOI:** 10.4102/sajp.v82i1.2405

**Published:** 2026-10-29

**Authors:** Thayananthee Nadasan, Oladapo Olagbegi, Simakahle Ntshembeni, Bongumenzi Ndwandwe, Siyethemba Mabaso, Laken Govender, Reina Abraham

**Affiliations:** 1 Department of Physiotherapy, College of Health Sciences, University of KwaZulu-Natal, Durban, South Africa; 2 Department of Clinical and Professional Practice, Nelson R. Mandela School of Medicine, University of KwaZulu-Natal, Durban, South Africa

**Keywords:** osteoarthritis, health-seeking behaviour, rehabilitation, physiotherapy, sub-Saharan Africa, scoping review

## Abstract

**Background:**

Osteoarthritis (OA) is a common musculoskeletal disorder associated with pain, functional limitation and disability. In sub-Saharan Africa, health-seeking behaviour (HSB) is influenced by beliefs, health literacy, access to services and healthcare systems, yet this evidence has not been synthesised.

**Objectives:**

To map and describe evidence on HSBs of people with osteoarthritis in sub-Saharan Africa, identify influencing factors, and highlight implications for physiotherapy practice and research.

**Method:**

A scoping review was conducted using the Arksey and O’Malley framework, guided by Levac et al. and the Joanna Briggs Institute population-concept-context approach. Searches were conducted from January 2000 to August 2026 in Google Scholar, PubMed, ScienceDirect and EBSCOhost-hosted databases using keywords and synonyms related to osteoarthritis, HSB and sub-Saharan Africa, combined with Boolean operators. Citation searching was also undertaken. Three reviewers independently screened titles, abstracts and full-text articles, while two reviewers independently extracted data. Findings were synthesised narratively using a socioecological framework.

**Results:**

Of 2710 records and one additional citation, five studies met inclusion criteria. Four were conducted in Nigeria and one in South Africa, all in rural settings. Health-seeking behaviour was largely delayed or fragmented. Influencing factors spanned intrapersonal, interpersonal, organisational and community domains. Key barriers included beliefs that osteoarthritis is a normal part of ageing, poor knowledge, limited person-centred care, weak referral pathways, high costs, long waiting times and poor access to rehabilitation.

**Conclusion:**

This review found very limited published evidence on the HSBs of people with osteoarthritis in sub-Saharan Africa. Available evidence suggests that delayed and fragmented help-seeking is influenced by interconnected individual, interpersonal, organisational and community factors, highlighting the need to strengthen access to timely, person-centred rehabilitation services.

**Clinical Implications:**

Delayed help-seeking and limited access to rehabilitation identified in this review highlight the need for physiotherapists to strengthen patient education, promote self-management, support timely referral pathways and expand community-based rehabilitation, particularly in underserved settings.

## Background

Osteoarthritis (OA) is the most common form of arthritis and one of the leading causes of chronic pain, activity limitation and long-term disability worldwide (Abhishek & Doherty 2013; Allen, Thoma & Golightly 2021). It affects more than 7% of the global population, with prevalence expected to increase because of ageing populations and rising obesity rates (Qiao et al. 2024). Although OA was historically described as a simple ‘wear-and-tear’ degenerative joint disorder, current understanding recognises it as a complex, multifactorial condition influenced by mechanical, metabolic, inflammatory and behavioural factors. These processes involve cartilage degradation, subchondral bone changes and low-grade inflammation, with risk factors including age, sex, obesity, genetics and biomechanical stressors (Berenbaum 2012; Palazzo et al. 2016; Yunus, Nordin & Kamal 2020).

Osteoarthritis commonly affects the knee, hip and hand, with knee OA accounting for over 60% of global cases (Long et al. 2022). The knee and hip are among the most commonly affected joints, and symptoms such as pain, stiffness and declining physical function often reduce participation in everyday activities and quality of life (Abhishek & Doherty 2013; Allen et al. 2021).

For people living with OA, outcomes are not determined by pathology alone. They are also shaped by how symptoms are recognised, interpreted and acted upon. Help-seeking for a chronic condition such as OA may involve decisions about whether care is needed, where to seek that care, when to seek it and which form of treatment is acceptable or affordable (Anwar, Green & Norris 2012; Mandlik, Jauhari & Vishwakarma 2024). These decisions are often influenced by beliefs, culture, knowledge, previous experiences, cost, transport, staff attitudes, availability of services and trust in the healthcare system.

In sub-Saharan Africa (SSA), these determinants of health-seeking behaviour (HSB) are especially important because access to healthcare is frequently constrained by poverty, geographical distance, limited rehabilitation services and overstretched public health systems (McLaren et al. 2023; Van der Hoeven et al. 2024). In such settings, patients may rely on family advice, pharmacies, traditional healers or delayed hospital attendance rather than timely engagement with formal care (James et al. 2018). This is particularly relevant to physiotherapy because conservative OA management depends heavily on education, exercise, self-management support and timely referral rather than on medication alone.

Despite the importance of HSB to OA outcomes, the available literature appears fragmented. Research from SSA has more often focused on OA prevalence, pain and disability than on how individuals with OA navigate care pathways or the reasons behind their treatment choices. A scoping review was therefore warranted to map the available evidence, identify what is currently known, what remains unclear and where future physiotherapy-focused research is needed.

The review question was: *What evidence exists on the health-seeking behaviours of people with osteoarthritis in sub-Saharan Africa?*

The specific objectives were:

- To map the available evidence on HSBs of people with OA in SSA.
- To evaluate factors influencing these behaviours.
- To determine how these factors affect health and health outcomes.
- To evaluate knowledge gaps and implications for future research, policy and rehabilitation practice.

## Method

### Study design

A scoping review design was adopted because the purpose of the study was to map the extent, range and nature of evidence rather than to assess intervention effectiveness. The review followed the methodological framework described by Arksey and O’Malley (2005), with consideration of later refinements by Levac, Colquhoun and O’Brien (2010). Reporting was structured in line with the Preferred Reporting Items for Systematic Reviews and Meta-Analyses (PRISMA) extension guidance used in contemporary scoping reviews (Page et al. 2021).

### Protocol and registration

A review protocol was not registered for this scoping review.

### Eligibility criteria

The review was guided by the Joanna Briggs Institute population-concept-context framework (Joanna Briggs Institute 2020).

#### Population

People of any age diagnosed with OA of the hip, knee, ankle and/or foot.

#### Concept

Health-seeking behaviours, including healthcare utilisation, treatment-seeking, health information-seeking and related care pathways.

#### Context

Countries in SSA.

Studies were included if they:

- were peer-reviewed
- used qualitative, quantitative or mixed methods designs
- were published in English or had an English translation
- were published between January 2000 and August 2026
- reported evidence on OA and HSB in SSA.

Studies were excluded if they:

- were not published in English
- were not conducted in sub-Saharan Africa
- did not involve adults with osteoarthritis of the knee, hip, ankle or foot
- did not report on HSB.

### Search strategy

The literature search was conducted between March 2025 and July 2025. The search was later extended to include literature published between July 2025 and August 2026. Electronic searches were undertaken in Google Scholar, PubMed, ScienceDirect and EBSCOhost-hosted databases to identify studies published between January 2000 and August 2026. Search terms combined keywords and synonyms related to osteoarthritis, HSB and sub-Saharan Africa using Boolean operators (AND/OR). Examples of search terms included osteoarthritis, osteoarthrosis, degenerative joint disease, HSB, healthcare seeking, treatment seeking, health service utilisation and the names of SSA countries. Citation searches of included studies were also undertaken to identify additional relevant articles.

### Study selection

All identified records were exported to Mendeley Reference Manager v2.134.0, where duplicates were removed. Three reviewers independently screened titles and abstracts, followed by full-text review of potentially eligible studies. Disagreements were resolved by a fourth reviewer. The selection process was summarised in a PRISMA flow diagram as illustrated in Figure 1.

**FIGURE 1:**
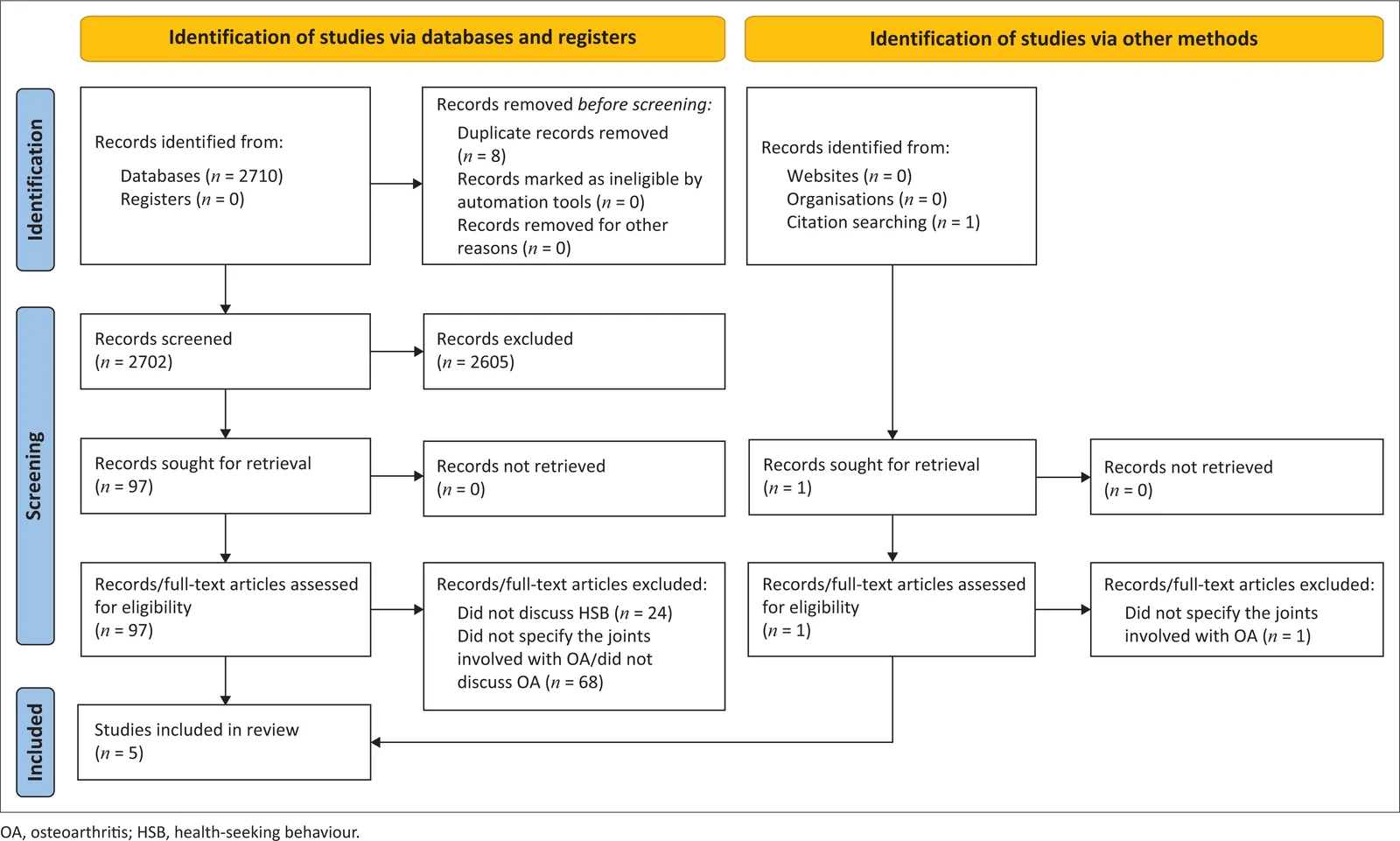
Preferred Reporting Items for Systematic Reviews and Meta-Analyses flow diagram depicting the scoping review process.

### Data charting and synthesis

The data extraction tool, developed and piloted in the scoping review protocol, was adjusted to fit the needs of the final study. Two reviewers independently extracted data using the piloted extraction form. Extracted data included study author, year, country, design, study aim, assessment tools, participant characteristics (age, sex and affected joint) and key findings relevant to HSB, as illustrated in Table 1 and Table 2. Extracted data were compared between the two reviewers and screened for discrepancies.

**TABLE 1:** Study characteristics.

Number	Source	Study location	Aims/objectives	Study design	Assessment tool(s)	Study findings
1	Akinpelu et al. (2011)	Nigeria	The prevalence and pattern of knee osteoarthritis in a rural community. To determine the health beliefs surrounding the causes of knee OA and the health-seeking behaviours.	Cross-sectional Study	Structured questionnaire American College of Rheumatology (ACR) classification for knee osteoarthritis Lequesne Algofunctional Index of Knee	Total OA prevalence is 36.9% in this population. Prevalence is higher among women and increase with age and body weight. Health seeking behaviours is poor in the community. Beliefs were significantly associated with health seeking behaviour.
2	Coetzee et al. (2020)	South Africa	To explore the rehabilitation needs of individuals with knee OA living in rural Western Cape.	Qualitative study	Semi-structured, face-to-face interviews. Open-ended questions	There is a need to address barriers in the rural PHC system to improve healthcare access and patient-centred care.
3	Faronbi et al. (2022)	Nigeria	To assess the pain characteristics, activity limitations and health-seeking behaviours of older adults with OA in Nigeria.	Cross-sectional study	Interviewer-administered questionnaires Chronic Pain Grade Scale (CPGQ) Western Ontario and McMaster Universities Arthritis Index (WOMAC) Healthcare Seeking Behaviour Questionnaire (HCSBQ)	Elder patients with OA have considerable pain and activity limitations. Patients are more likely to exhibit negative health seeking behaviours with the intention of relieving pain.
4	Ibebuike and Peters (2024)	Nigeria	To investigate the prevalence of osteoarthritis among people of Ogbaku Kingdom in Imo State, Nigeria. To investigate the utilisation of health care services among people suffering from osteoarthritis in Ogbaku Kingdom. To investigate the relationship between some socio-demographic characteristics and utilisation of healthcare services among people suffering from osteoarthritis in Ogbaku Kingdom.	Cross-sectional study	Clinical assessment using the American College of Rheumatology (ACR) clinical criteria, followed by a pretested questionnaire on healthcare utilisation and clinical history.	OA is prevalent among the elderly. Moderate use of healthcare services was noted among the elderly with OA. Pharmacotherapy was the most utilised service, compared to in-patient and out-patient services. Age, education and occupation were significantly associated with healthcare usage.
5	Owoyemi et al. (2025)	Nigeria	To explore the current experiences of care and living with osteoarthritis and/or joint pain, unmet needs and care gaps in the management of people living with osteoarthritis in Nigeria. To identify barriers and facilitators of care from patient perspectives.	Qualitative study	Focus groups	Patients have single or multiple joint pain, which poses a burden for everyday living. Various socio-economic factors were identified as barriers and/or facilitators for healthcare usage.

Note: Please see the full reference list of the article, Nadasan, T., Olagbegi, O., Ntshembeni, S., Ndwandwe, B., Mabaso, S., Govender, L. et al., 2026, ‘Health-seeking behaviours of people with osteoarthritis in sub-Saharan Africa: A scoping review’, *South African Journal of Physiotherapy* 17(1), a2405. https://doi.org/10.4102/sajp.v17i1.2405, for more information.

PHC, primary healthcare; OA, osteoarthritis.

**TABLE 2:** Participant characteristics.

Number	Source	Participants	Size and sex						Age (years)	Mean age (years)	Joint(s) affected				
			Total			With OA									
			Female (%)	Male (%)	*n*	Female (%)	Male (%)	*n*			Knee (%)	Hip (%)	Foot (%)	Ankle (%)	*n*
1	Akinpelu et al. (2011)	Adults ≥ 30 years	10.5	89.5	1403	25.8	74.2	229	≥ 30	43.9 ± 121.8	100	-	-	-	229
2	Coetzee et al. (2020)	Adults ≥ 45 years diagnosed with knee osteoarthritis	93.75	6.25	16	-	-	-	≥ 45	Unknown	100	-	-	-	16
3	Faronbi et al. (2022)	Older adults ≥ 60 years living with osteoarthritis	86.5	13.6	230	-	-	-	60–99	76.37 ± 11.23	73	7.8	-	11.7	230
4	Ibebuike and Peters (2024)	Adults ≥ 40 years with osteoarthritis	60.79	39.21	1349	72.96	27.04	392	≥ 50	Unknown	100	-	-	-	392
5	Owoyemi et al. (2025)	Adults ≥ 45 years with osteoarthritis or who sought care for joint pain	18.60	81.40	30	60	40	18	≥ 45	63.8	43.3	13.3	16.5	-	18

Note: Please see the full reference list of the article, Nadasan, T., Olagbegi, O., Ntshembeni, S., Ndwandwe, B., Mabaso, S., Govender, L. et al., 2026, ‘Health-seeking behaviours of people with osteoarthritis in sub-Saharan Africa: A scoping review’, *South African Journal of Physiotherapy* 17(1), a2405. https://doi.org/10.4102/sajp.v17i1.2405, for more information.

OA, osteoarthritis.

A narrative synthesis was undertaken. To strengthen interpretation, the findings were organised using a modified socioecological framework adapted from McLeroy et al. (1988), with themes grouped into intrapersonal, interpersonal, organisational, community and public policy domains, as illustrated in Table 3.

**TABLE 3:** Health-seeking behaviours.

Domain	Sub-domain	Reference	Behaviour type	Sources
Intrapersonal	Beliefs	OA is normal with aging Injuries cause OA	Negative Negative	Akinpelu et al. (2011) Ibebuike and Peters (2024); Owoyemi et al. (2025)
	Knowledge	Poor knowledge relating to OA and management options	Negative	Akinpelu et al. (2011); Coetzee et al. (2020); Owoyemi et al. (2025)
	Pain and functional limitations	Often seek care only when symptoms worsen	Negative	Akinpelu et al. (2011); Faronbi et al. (2022)
	Preference	Prefer specific care/treatments	Negative	Akinpelu et al. (2011); Coetzee et al. (2020)
Interpersonal	Patient care	Lack of patient-centred care	Negative	Coetzee et al. (2020)
	Knowledge	Uninformed health practitioners	Negative	Coetzee et al. (2020); Owoyemi et al. (2025)
Organisational	Referral system	Referred to outpatient facilities for management	Positive	Ibebuike and Peters (2024)
		Poor referral systems	Negative	Coetzee et al. (2020)
	Costs	Hospital expenditure	Negative	Faronbi et al. (2022); Owoyemi et al. (2025)
	Waiting times	Long waiting times	Negative	Coetzee et al. (2020); Owoyemi et al. (2025)
Community	Accessibility	Lack of accessibility in rural areas	Negative	Akinpelu et al. (2011); Faronbi et al. (2022); Owoyemi et al. (2025)
	Resource poor and understaffing	Unable to send necessary health staff to rural areas	Negative	Coetzee et al. (2020); Owoyemi et al. (2025)
	Participation	Functional limitations leading to social isolation	Negative	Coetzee et al. (2020)

Note: Please see the full reference list of the article, Nadasan, T., Olagbegi, O., Ntshembeni, S., Ndwandwe, B., Mabaso, S., Govender, L. et al., 2026, ‘Health-seeking behaviours of people with osteoarthritis in sub-Saharan Africa: A scoping review’, *South African Journal of Physiotherapy* 17(1), a2405. https://doi.org/10.4102/sajp.v17i1.2405, for more information.

OA, osteoarthritis.

### Critical appraisal of sources of evidence

A formal critical appraisal of included studies was not conducted, as the aim of this scoping review was to map the available evidence rather than to assess methodological quality. This approach is consistent with established scoping review methodology.

### Ethical considerations

Ethical clearance to conduct this study was obtained from the Humanities and Social Sciences Research Ethics Committee (HSSREC), University of KwaZulu-Natal (No. HSSREC/00029785/2025).

## Results

### Study selection

A total of 2710 records were identified through database searches, with one additional record identified through citation searching, yielding 2711 records. After the removal of eight duplicates, 2703 records were screened. Of these, 2605 records were excluded during title and abstract screening as they did not meet the inclusion criteria, most commonly because they did not address HSB, were not specific to osteoarthritis of the eligible joints or were conducted outside the sub-Saharan African context. This resulted in 98 full-text articles being assessed for eligibility. Ultimately, five studies met the inclusion criteria and were included in the review (Figure 1).

### Characteristics of included studies

Of the five included studies, four were conducted in Nigeria (Akinpelu et al. 2011; Faronbi et al. 2022; Ibebuike & Peters 2024; Owoyemi et al. 2025) and one in South Africa (Coetzee, Giljam-Enright & Morris 2020). Table 1 provides a summary of the study characteristics. Two studies used qualitative designs (Coetzee et al. 2020; Owoyemi et al. 2025) and three used cross-sectional quantitative designs (Akinpelu et al. 2011; Faronbi et al. 2022; Ibebuike & Peters 2024). All five studies were undertaken in rural or low-resource settings.

The participants were predominantly community-dwelling adults diagnosed with osteoarthritis of the hip, knee, ankle and/or foot. Across the studies, participants with OA were predominantly women over 45 years of age. Although Akinpelu et al. (2011) recruited a predominantly male community sample, the prevalence of osteoarthritis was higher among women. This study also included adults aged 30 years and older, whereas the remaining studies primarily included participants aged 45 years or older. The knee was the most affected joint, followed by the hip, ankle and foot. Table 2 summarises the participant characteristics.

### Thematic findings

The following themes are the findings from all five studies, which are also summarised and displayed in Table 3.

### Intrapersonal factors

All five studies identified intrapersonal factors that shaped HSB. A recurring finding was the belief that OA is a normal or inevitable part of ageing, which reduced urgency to seek formal care (Akinpelu et al. 2011; Ibebuike & Peters 2024; Owoyemi et al. 2025). Some participants also associated OA with previous injury and were less likely to seek help for joint pain in the absence of trauma (Owoyemi et al. 2025).

Poor knowledge of OA and its management was another prominent theme. Limited understanding of the chronic nature of OA, uncertainty about treatment options and low health literacy contributed to delayed help-seeking and reliance on self-treatment or informal care (Akinpelu et al. 2011; Coetzee et al. 2020). In some studies, patients sought care only when pain became severe or when physical function was substantially impaired (Akinpelu et al. 2011; Faronbi et al. 2022).

Participants also expressed preferences for specific types of care. Some were dissatisfied with receiving medication alone and wanted more specialised or rehabilitative care, including better explanations and alternative management options (Coetzee et al. 2020; Faronbi et al. 2022).

### Interpersonal factors

Two studies highlighted the influence of healthcare providers on help-seeking (Coetzee et al. 2020; Owoyemi et al. 2025). Patients described receiving conflicting or inadequate explanations about OA, which increased confusion and reduced confidence in formal care. Poor communication, limited patient education and a lack of person-centred engagement discouraged ongoing attendance.

These findings are highly relevant to physiotherapy because they suggest that communication quality and consistent patient education are not peripheral issues; they directly shape whether people return for care, adhere to advice and understand OA as a manageable condition rather than an untreatable consequence of ageing.

### Organisational factors

Four studies described organisational barriers to care-seeking (Coetzee et al. 2020; Faronbi et al. 2022; Ibebuike & Peters 2024; Owoyemi et al. 2025). Poor referral systems were a major issue. While some patients were referred to outpatient or pharmaceutical services, referrals for rehabilitation, especially physiotherapy, were inconsistently reported and sometimes absent.

Cost was another major barrier. Consultation fees, transport costs and broader out-of-pocket expenses discouraged hospital attendance and made informal care options more attractive (Faronbi et al. 2022; Owoyemi et al. 2025). Long waiting times, short consultations and service inefficiencies also shaped negative experiences of formal care (Coetzee et al. 2020; Owoyemi et al. 2025).

### Community factors

Community-level factors were strongly represented. All included studies were conducted in rural or under-resourced environments where access to healthcare facilities was limited. Participants reported long travel distances, scarce services, understaffing and limited availability of healthcare professionals (Coetzee et al. 2020; Faronbi et al. 2022; Ibebuike & Peters 2024; Owoyemi et al. 2025). In these settings, local pharmacy stores (chemists), where medications could be purchased, and traditional healers were often more accessible than formal healthcare facilities.

Some findings also suggested that OA-related functional limitation contributed to reduced participation and social isolation, which may further constrain help-seeking and continuity of care.

### Public policy

No included study explicitly addressed the influence of public policy or municipal structures on OA-related HSB. This absence represents a significant evidence gap.

## Discussion

This scoping review mapped and synthesised the available evidence on the HSB of patients with OA in SSA. Only five studies met the eligibility criteria, which, rather than reflecting a methodological limitation, highlights the limited volume of research that explicitly examines OA-related HSB within a clearly defined SSA context. This finding represents an important evidence gap rather than a limitation of the review methodology, suggesting that HSB in OA remains under-researched in the region. While broader literature may address musculoskeletal pain, disability or healthcare access, few studies directly explore how individuals with OA recognise symptoms, make decisions about care and navigate available health systems.

Across the included studies, HSB was consistently described as delayed, fragmented and largely negative. These behaviours were not random but reflected complex and interrelated influences across the intrapersonal, interpersonal, organisational and community domains. The findings suggest that the HSBs of people with OA are shaped by cumulative experiences and contextual constraints rather than by isolated decisions, reinforcing the importance of adopting a socioecological perspective when interpreting health behaviours (McLeroy et al. 1988).

At the intrapersonal level, beliefs and knowledge emerged as central determinants of HSB. A dominant theme across the included studies was the normalisation of OA as an inevitable consequence of ageing, leading many individuals to perceive the condition as untreatable or not requiring medical attention (Akinpelu et al. 2011; Ibebuike & Peters 2024; Owoyemi et al. 2025). Some participants also believed that osteoarthritis was primarily caused by previous injury and were therefore less likely to recognise persistent joint pain in the absence of trauma as requiring medical assessment. These beliefs contributed to delayed engagement with formal healthcare and greater reliance on informal care pathways, including local pharmacy stores (chemists) and traditional healers. Poor health literacy further compounded these behaviours, as many individuals lacked an adequate understanding of OA, its progression and available management options (Coetzee et al. 2020). Limited access to reliable health information in rural settings, where communities often depend on informal sources such as family members, the media and local networks, may reinforce misconceptions and perpetuate negative HSBs (Anasi 2012; Emmanuel & Ekoja 2020; McClintock et al. 2019; Oladele et al. 2019).

These findings highlight the importance of reframing osteoarthritis as a manageable chronic condition rather than an inevitable consequence of ageing through improved patient education and consistent, evidence-based communication by healthcare providers.

Importantly, delayed care-seeking was frequently symptom driven. Patients often sought care only when pain became severe or when functional limitations significantly interfered with daily activities (Akinpelu et al. 2011; Faronbi et al. 2022). While pain acted as a motivator to seek help, it also highlighted missed opportunities for early intervention, particularly given that conservative management strategies such as education, exercise and self-management are most effective when introduced early.

Interpersonal and organisational factors further influenced HSB, revealing that patient behaviour is closely linked to interactions with healthcare systems and providers. Several studies reported that healthcare practitioners provided limited, inconsistent or conflicting information about OA, which created confusion and reduced patient confidence in formal healthcare services (Coetzee et al. 2020; Owoyemi et al. 2025). In some cases, practitioners appeared to have limited disease-specific knowledge or relied on outdated explanations, such as describing OA as ‘wear-and-tear’, which may reinforce patient misconceptions (Rahmati et al. 2017). The lack of person-centred care and perceived disinterest from healthcare providers contributed to dissatisfaction, discouraging patients from returning for follow-up care.

These findings underscore a critical gap in healthcare providers’ knowledge of osteoarthritis and in the communication of evidence-based information to patients. Health professionals play a central role in shaping patient understanding and engagement, and inadequate or poorly communicated information can inadvertently reinforce misconceptions and negative HSBs. Emerging evidence from SSA suggests a need for context-specific, guideline-informed OA care and improved training for healthcare providers to support consistent and evidence-based management (Adetunji et al. 2025; Babatunde et al. 2025).

Organisational barriers were also prominent. Poor referral systems, particularly limited referral to physiotherapy and rehabilitation services, were identified as significant contributors to negative HSB. Management approaches were frequently centred on pharmacological treatment, with limited referral to rehabilitation services and little emphasis on exercise therapy or long-term self-management strategies. Consequently, patients often received care that did not meet their expectations, contributing to frustration and disengagement from formal healthcare (Coetzee et al. 2020; Owoyemi et al. 2025). Poor multidisciplinary collaboration and limited awareness of the role of physiotherapy may further explain these referral gaps (Olaleye & Abiodun 2022).

Cost and accessibility were additional barriers that significantly influenced behaviour. High consultation fees, transport costs and long waiting times discouraged patients from accessing healthcare facilities, particularly in rural and low-income settings (Faronbi et al. 2022; Owoyemi et al. 2025). Overburdened health systems, characterised by understaffing and resource constraints, created environments in which patients experienced long delays and short, unsatisfactory consultations. These experiences contributed to distrust in formal healthcare and encouraged the use of alternative care options.

At the community level, limited availability of healthcare services in rural areas further shaped HSB. Patients often had to travel long distances to access care, and healthcare facilities were frequently understaffed or lacked essential resources. In this context, reliance on local pharmacies (chemists), where medications could be purchased, and on traditional healers was not simply a cultural preference but a pragmatic response to limited access and affordability. These findings highlight that so-called ‘negative’ health behaviours may, in reality, represent rational decision-making within constrained environments.

The widespread use of informal care pathways raises important concerns. While these options are more accessible and affordable, they may contribute to inappropriate self-medication, delayed diagnosis and suboptimal management. In vulnerable populations, such as older adults with functional limitations, these practices may increase the risk of adverse outcomes, including medication misuse and dependency (Perrot et al. 2019). At the same time, these findings suggest an opportunity for integrating community-based providers, such as pharmacists and traditional healers, into broader health education and referral strategies.

The review also highlights the underrepresentation of physiotherapy in OA management pathways within SSA. Evidence-based guidelines emphasise the importance of exercise, education, weight management and self-management as first-line interventions for OA. However, limited referral to physiotherapy services suggests that these approaches are not consistently implemented. This gap may contribute to worsening symptoms, reduced function and decreased quality of life, as well as increased reliance on pharmacological treatments (Coetzee et al. 2020; Kareem et al. 2024). Furthermore, the progression of OA symptoms has been associated with psychological consequences, including depressive symptoms and social isolation, which may further reduce engagement with healthcare services and perpetuate a cycle of poor health outcomes (Coetzee et al. 2020; Vina et al. 2019).

Collectively, these findings demonstrate that HSB in OA is shaped by systemic and structural factors as much as individual beliefs. Pain may drive patients to seek care initially (Ali, Walsh & Kloseck 2018), but inadequate education, limited treatment options, poor service experiences and financial barriers often drive them away. Improving HSB, therefore, requires a shift from a purely biomedical model to a more integrated, patient-centred and context-responsive approach.

The findings of this review have important implications for clinical practice and health policy. The predominance of medication-centred care, together with limited referral to physiotherapy and rehabilitation services, suggests opportunities to strengthen OA management within primary healthcare. Primary healthcare providers could enhance care by incorporating structured patient education, exercise prescription and self-management support alongside pharmacological management. At a policy level, strengthening primary healthcare systems, improving access to rehabilitation services and promoting multidisciplinary models of care may facilitate more comprehensive OA management. Task-shifting strategies, such as training community health workers to deliver basic OA education and support, may further improve access to care in underserved areas.

This scoping review mapped and synthesised the available evidence on the HSBs of people with osteoarthritis in sub-Saharan Africa. Only five studies met the eligibility criteria, highlighting a substantial evidence gap. Existing research is largely limited to rural communities in Nigeria and South Africa, with little evidence from other countries, urban populations or different healthcare settings. This limited geographical and contextual representation restricts understanding of how HSBs may vary across the region and underscores the need for further research.

Future research should therefore examine HSBs across a broader range of contexts and evaluate scalable, context-specific interventions to improve timely access to care. There is also a need to evaluate the effectiveness of community-based programmes, including group exercise, patient education and peer-support initiatives, in improving both health outcomes and HSBs.

By aligning healthcare services with patient needs through accessible, affordable and evidence-based interventions, health systems in sub-Saharan Africa can improve timely access to care, strengthen rehabilitation services and enhance the quality of life for people living with osteoarthritis.

### Limitations

This review has several limitations. Firstly, only five studies met the eligibility criteria, which limits the breadth of available evidence. However, this limited yield is also a key finding, as it highlights the scarcity of disease-specific research on OA-related HSB in SSA.

Secondly, the review included only peer-reviewed articles published in English. Relevant studies in other languages or in grey literature may therefore have been missed.

Thirdly, although a comprehensive search strategy was undertaken across multiple databases without restricting the search to specific countries within sub-Saharan Africa, the included studies were limited to Nigeria and South Africa and were all conducted in rural settings. This reflects the current paucity of published evidence rather than a limitation of the search strategy. Consequently, the findings may not fully represent HSBs in other SSA countries or urban settings. In addition, only English-language publications were included, which may have excluded relevant studies published in other languages commonly used within the region.

### Recommendations

Future research should prioritise:

- primary studies on OA-related HSB in underrepresented SSA countries and urban settings
- context-specific investigations into referral pathways to physiotherapy and other rehabilitation services
- the development and evaluation of patient education interventions that address misconceptions about OA
- implementation research examining the uptake of guideline-informed OA care within primary healthcare and community settings
- studies exploring health policy, service planning and access to rehabilitation services for people with OA.

The findings of this review also have important implications for clinical practice and health service planning. Health systems should strengthen multidisciplinary osteoarthritis care pathways by promoting earlier referral to physiotherapy and other rehabilitation services, improving community education to address misconceptions about osteoarthritis and expanding accessible, responsive rehabilitation services, particularly in rural and under-resourced settings.

### Clinical implications

The findings of this review have several important implications for physiotherapy practice. Physiotherapists should play a more active role in the early education of patients with OA, particularly regarding pain mechanisms, disease prognosis, exercise therapy and self-management strategies. Early education may help to address misconceptions about OA and encourage more timely and appropriate HSBs.

The review also highlights the need to strengthen the integration of physiotherapy within referral pathways, particularly at the primary healthcare level and in rural settings where access to rehabilitation services is often limited. Earlier referral to physiotherapy may facilitate timely, evidence-based interventions, promote self-management and reduce reliance on pharmacological management alone. Strengthening multidisciplinary referral pathways may therefore improve access to comprehensive OA care and enhance patient outcomes.

In addition, physiotherapists should strengthen and expand context-sensitive rehabilitation approaches, including home-based and community-based programmes, particularly in low-resource settings. Expanding access to these services may help overcome barriers such as transport costs, long travel distances and limited availability of rehabilitation services, thereby improving access to ongoing osteoarthritis management.

Physiotherapy services should further prioritise person-centred communication, as the review identified poor communication and unmet patient expectations as important contributors to dissatisfaction with formal healthcare. Clear, consistent and culturally appropriate communication may strengthen patient understanding, build trust and encourage continued engagement with rehabilitation and other recommended healthcare services.

Finally, physiotherapists have an important advocacy role to play in service design and policy development. By contributing to discussions on healthcare planning and resource allocation, physiotherapists can help ensure that OA management in sub-Saharan Africa moves beyond medication-centred models towards more comprehensive, rehabilitation-informed and patient-centred care.

## Conclusion

This scoping review found very limited published evidence on the HSBs of people with OA in SSA. The five included studies suggest that help-seeking is influenced by interconnected intrapersonal, interpersonal, organisational and community factors. Beliefs that OA is a normal part of ageing, poor knowledge of management options, inconsistent communication from providers, weak referral systems, cost barriers, long waiting times and poor rural access to services all contribute to delayed or fragmented care.

The small number of included studies should be interpreted as evidence of a substantial knowledge gap. There is a clear need for more context-specific research and stronger integration of rehabilitation, particularly physiotherapy, into OA care pathways across SSA.
